# Integrated Method of Teaching in Web Quest Activity and Its Impact on Undergraduate Students’ Cognition and Learning Behaviors: A Future Trend in Medical Education

**DOI:** 10.5539/gjhs.v7n4p249

**Published:** 2015-01-14

**Authors:** Zohreh Badiyepeymaie Jahromi, Leili Mosalanejad

**Affiliations:** 1 Nursing Department, Jahrom University of Medical Sciences, Jahrom, Iran; 2 Mental Health Department, Jahrom University of Medical Sciences, Jahrom, Iran

**Keywords:** Web Quest, problem solving, self-directed learning, self-regulated learning

## Abstract

**Introduction:**

Web Quest is one of the new ways of teaching and learning that is based on research, and includes the principles of learning and cognitive activities, such as collaborative learning, social and cognitive learning, and active learning, and increases motivation. The aim of this study is to evaluate the Web Quest influence on students’ learning behaviors.

**Materials and Methods:**

In this quasi-experimental study, which was performed on undergraduates taking a psychiatric course at Jahrom University of Medical Sciences, simple sampling was used to select the cases to be studied; the students entered the study through census and were trained according toWeb Quest methodology. The procedure was to present the course as a case study and team work. Each topic included discussing concepts and then patient’s treatment and the communicative principles for two weeks. Active participation of the students in response to the scenario and introduced problem was equal to preparing scientific videos about the disease and collecting the latest medical treatment for the disease from the Internet. Three questionnaires, including the self-directed learning Questionnaire, teamwork evaluation Questionnaire (value of team), and Buffard self-regulated Questionnaire, were the data gathering tools.

**Results:**

The results showed that the average of self-regulated learning and self-directed learning (SDL) increased after the educational intervention. However, the increase was not significant. On the other hand, problem solving (P=0.001) and the value of teamwork (P=0.002), apart from increasing the average, had significant statistical values.

**Conclusions:**

In view of Web Quest’s positive impacts on students’ learning behaviors, problem solving and teamwork, the effective use of active learning and teaching practices and use of technology in medical education are recommended.

## 1. Introduction

Nowadays, the development of human knowledge and awareness in all fields has caused traditional teaching and learning methods to lose their efficacy, so in order to keep pace with the changing world, one should look for new ways to increase and transfer knowledge (Bowles, 2000). In medical education, due to the complexity that accompanies theoretical and practical training, this issue is of greater importance. And educational planners and administrators need to adopt new strategies by using appropriate educational methods to empower students to enter the health care system (Dent & Harden, 2013).

Web Quest is web-based activities of teaching and learning that use learning skills that involve high levels of thinking (Bernie Dodge, 1995). Web Quest includes the principles of learning and cognitive activities, such as cooperative learning, learning framework, problem solving, formative thinking and learning, real and objective evaluation, cognitive and social learning, active learning and increasing motivation (Karimi Moonaghi & Armat, 2013). Web Quest is based on Constructivist Learning Theory. According to the structural approach, it’s the learner who is responsible for higher learning, not the trainer, and knowledge is built up by him in his mind. Some of the principles of web-based learning are retrieval and imagination, classification and generalization, comparison and evaluation, analysis and synthesis, inference and deduction; so students are required to answer some questions, solve some problems, or elucidate a set of observations that have been provided for them. If applied effectively, this method can help students learn how to form questions in a good way, identify and collect appropriate evidence, conduct a systematic study, analyze and interpret the results, draw conclusions and evaluate values and the magnitude of the results (Mohammadi Azizabadi, 2010). Today, through out the world, tens of thousands ofteachershave accepted Web Quest as an appropriate way of using the Internet that involves the students in a way of thinking that is essential in the 21st century(Bernie & Dodge, 2007).

One of the advantages of Web Quest is that each learner is able to make progress at his own speed by using self-direction (Schwarz, 2014). In terms of process and in terms of personal characteristics, this kind of learning activity best with self-directed learning. In this method of learning, the student is responsible for controlling the further education that causes students to achieve high levels of self-direction to succeed in the learning environment (L. Song & Hill, 2007). Self-directed learners are active and spontaneous persons who take the initiative in learning, instead of passively waiting for learning. Self-direction is apsychological state in which the learner feels that he/she is responsible for his/her own learning (Piskurich, 2004). Self-directed learning is one of the most famous educational methods in the humanistic approach. In humanistic theory, the learning focus is on individual needs whose final goal is self-actualization and self-discovery. Learning that is rooted in this approach has several features, among them personal involvement of the learner, which is a form of learning in which the learner is the starter and learning evaluation is done by the learner himself/herself. All of these features lead to the development of an autonomous learner. Humanistic theory is theory of motivation, and the essence of motivation for the learner is the desire to achieve what he/she is able to achieve (Doosti Irani, Abolhasani, & Haghani, 2012).

After graduation, medical students are expected to know about self-directed learning inprofessional life, and have an awareness of their weaknesses in knowledge and problem-solving fields; so the educational and organizational environments have seriously emphasized the importance of self-oriented learning and considered it as a necessary skill in training and work inthe21st century (Yousefy & Gordanshekan, 2010).

Research has shown that the simultaneous introduction of various information resources and instructional methods, on various teaching subjects, will lead to more effective teaching and increase the students ’ability to learn and their satisfaction with teaching methods(Jang, Kim, & Park, 2005; Sung, Kwon, & Ryu, 2008).

Case Based Learning (CBL), an educational method, is an effective method of training doctors and nurses in America, Europe and Australia (Chan, Hsu, & Hong, 2008; Dietrich, De Silva, & Young, 2010; Thurman, Volet, & Bolton, 2009). Learning through case study is an educational method that facilitates students’ learning in a student-centered context; it is a series of real or simulated clinical problems that prepares tudents for professional problems (Kaddoura, 2011). It is a working model and a training strategy that will enhance students’ ability to solve a clinical problem (Morrow, 2009), and can boost students’ motivation and establish are a learning experience for them (Carroll & Borge, 2007).

Team learning is also an important teaching strategy in nursing education programs; it causes students to learn social skills, fosters a spirit of cooperation and enhances their professional skills (Xyrichis & Ream, 2008). Apart from improving students’ understanding, team teaching methods increase the possibility of the retention of the content, critical thinking, positive attitudes toward the course and students ’engagement with their learning, which plays a major role in their learning and in improving their final scores (Clark, Nguyen, Bray, & Levine, 2008). A combination of these benefits with those of WebQuest activity —the created synergy can greatly enhance students’ learning.

What is definitely derived from searching the available resources is that the concept ofWeb Quest in our countryis an unknown one, not only in general education, but also in academic training(Karimi Moonaghi & Armat, 2013), and few studies have been conducted on this training tool and its consequences. Furthermore, it is noteworthy that the use of active teaching and learning practices in many developing countries, including Iran, is in an inchoate state, and, although education I moving towards the use of active methods, any kind of educational reform, when compared with the traditional and in active practices, faces resistance and numerous challenges. On the other hand using combined methods of teaching and learning with all the mentioned cases correlates with preparing educational context, student admittance and teacher management. So, according to the afore mentioned reasons, and the difficulty of training courses on mental illnesses and the fact that many of the signs and symptoms and how to deal and communicate with the patients are the difficult parts of this field, approaches that can place the student in the middle of the problem, encourage him/her to think, and provide a better understanding of concepts are becoming more and more popular. This will make greater and steadier learning possible for students, and create a fun learning environment. So, the researchers sought to study the impact of an integrative method of teaching on self-oriented learning, the value of teamwork, and the level of students’ satisfaction with the educational model.

## 2. Material and Methods

In this quasi-experimental study, which was conducted on students at Jahrom University of Medical Sciences, simple sampling was used for selection, and the students were trained according to the integrated method (a case study based and team-learning with Web Quest activities).

### 2.1 Study Design

The students who had mental health and psychiatric course were selected for this study through the census method; next, the teacher presented various facts about the illnesses in a fashion that at one session, the general concepts of the course, and at the next session a continuation of the previous discussion, in relation to healing and the therapeutic approach (psychosocial), would be discussed. At the beginning of the first session, which lasted1 hour, topics, such as an introduction of the disease, epidemiology, its types, a detailed discussion about the symptoms, and the basic principles of the lesson were fully presented. At the end of the first session, multiple-choice questions were submitted (4 questions each time) and answered in a teamwork fashion. The purpose of these questions, called the individual and group assurance tests, was to ensure that the subject matter had been learned. At the end of the session—10 to15minutes—the videos collected by the groups about thedisorder and related symptoms would be played. Each session topic was introduced to the students through the lesson plan, and the instructor would remind the groups about the next week topic so that they could prepare related videos, and then 4 short case studies of the disease would be mentioned and presented by raising a problem about aspects related to patient care, communication with patients and other therapeutic nursing-care relatedissues in slides. Each group performed teamwork for 1 hour to find answers to the problem. Raised questions were about therapeutic communication techniques with patients and were designed for each level of the disease separately. Then the students would initiate a group discussion for problem solving. The participants were divided into 4 groups of 10 people, based on their student I.D. numbers. Next, a title was chosen for each group, and the results of the teamwork were considered as assessing scenarios and case studies for the overall group’s score. Regarding its motivational nature, this score had no impact on the students’ final scores, but the students were told that active participation in the group activities of the class had an equivalent score to that of quizzes. Then, a representative for each group was elected by the members of the group every two sessions. In this program, this representative described the group members’ activities regarding medicinal treatments for each specific category of disease, in the form of an overview of introduced Internet sites, such as Up-to-date, for the whole class. Due to lack of time, medicinal treatments were introduced as a general category of commonly used drugs for the diseases from valid websites, and a maximum of 15 minutes was allocated to them. So the students’ Web Quest activities consisted of collecting appropriate videos about the diseases and the medicinal treatments for the disease groups.

In this research, the self-directed learning questionnaire, self-regulation questionnaire, problem solving inventory s (PsI), and the value of team work questionnaire(value of team) were used.

Guglielmino’s self-directed learning readiness scale (SDLRS) is a self-report questionnaire with 41 items that are of five-part Likert (ranging from “always” to “hardly ever”) and cover the three areas of self-management, desire for learning, and self-control. Internal consistency of the Questions and test-retest reliability of the test were calculated at 0.95 and 0.68, respectively. Scores were calculated out of100 for each area and scores of less than33.3 were considered as low, between33.3 and 66.7 as average, and more than that as too much. The Cronbach’s alpha of the sub-scales of self-management, learning tendency and self-control were 0.81, 0.78, and 0.84, respectively (Fisher, King, & Tague, 2001). This questionnaire was conducted by Sajadian and Nadion 1135 medical and dental students in2012 and its validity and reliability were confirmed (Nadi & Sadjadian, 2011). The maximum and minimum scores of this tool are 205 and 41, respectively.

**Study design**


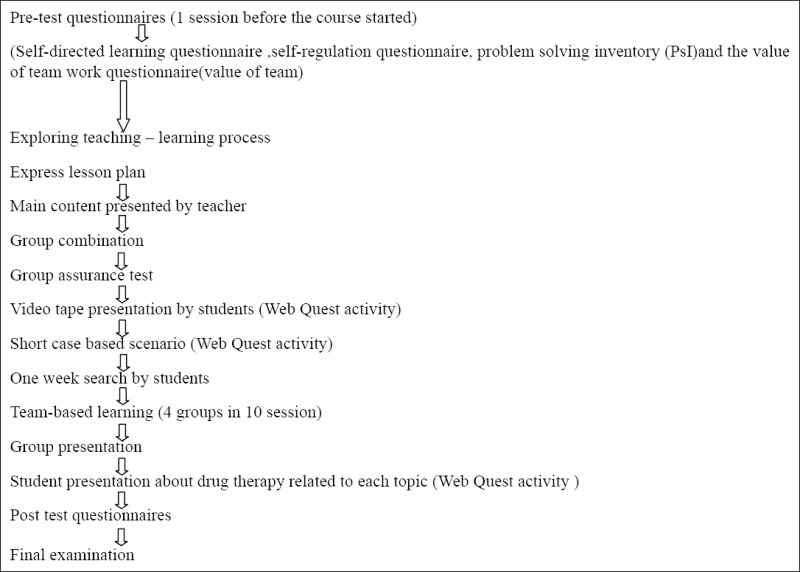


The second questionnaire–14 questions is a self-regulation questionnaire, designed by Bouffard et al. (1995) (Bouffard, Boisvert, Vezeau, & Larouche, 1995), and standardized by Kadivar (2001). The overall reliability of the questionnaire has been 0.71 on the basis of the Cronbach alpha. The subscalevalidity of cognitive strategiesandmeta-cognitivewere reported to be 0.70and 0.68, respectively. Regarding its structure, item analysis indicated that the correlation coefficient of the questions was suitable and that assessment tools consisted of two components. Construct validity of this questionnaire has been in an acceptable range and this tool can explain the self-regulation variance of 0.52. The validity of the structure has been optimal. In this test, 5 options are considered for each question: “strongly agree; agree; no idea; disagree; and strongly disagree,” each rated 1 to 5, respectively. Grading of the questions 5, 13 and 14 was contrary to that of the rest (Kadivar, 2001). The problem-solving inventory is the third questionnaire used. Problem-solving questionnaire, with 35 items, was invented by Heppner and Petersen (1982) for the assessment of respondents’ understanding of their problem-solving behaviors and for measuring how people react to the issues of their daily activities (PS). The problem-solving questionnaire that is based on the rotation factor analysis has 3 separate scales: Confidence in resolving problems with 16 items, avoidance style with 16 items, and personal control with 16 items. This questionnaire is based on 6 levels of the Likert scale, with low scores indicating high awareness levels of problem-solving abilities (1 = totally agree, 2 = moderately agree, 3 = slightly average, 4 = slightly disagree, 5 = moderately disagree, 6 = totally disagree). To prevent abuse in answering, 15 items have been stated with negative verbs (graded reversely). The total score of the questionnaire is calculated by adding up the scores of all replies (Heppner, Witty, & Dixon, 2004). This questionnaire was translated by Rafati&Khosravi in 1996 and was used in Iran for the first time.

The teamwork value questionnaire (VTs) has been developed in 17 items by (Haidet et al., 2002) in a 5-part scale ranging from “totally agree” to “totally disagree” and includes two subscales: team work and working with classmates. Cronbach’s Alpha coefficient for the entire questionnaire has been from 0.79 to 0.81 (Haidet, O’Malley, & Richards, 2002). It has been standardized in Iran and will be used later on. The questionnaires were completed in two steps: before and after the use of Web Quest activities. Also, to help students to improve their learning and retrieving, the tests (group assurance tests) were used and the students’ satisfaction and the effects of the method on students were studied through essay questions. Also, adding the qualitative analysis part represented the article as a quantitative-qualitative (mixed) one. For the reliable completion of the questionnaires, 2 separate sessions, each lasting thirty-minutes, were held.

## 3. Results

Of the 43 students who participated in the study, 23 were female. Most of them were in the age range of 22-24 years, and 4 were married.

Data distribution showed that a large percentage of students in the post-test scored high on the self directed learning subscales. There were not any significant differences within group pretest-posttestresults (Table 1).

**Table 1:** Mean difference between directed-learning and self-regulated learning within groups before & after intervention

		Self-directed learning		
		Subscale		

		Self-control	Self-engagement	Self-management
Integrated Web Quest method	Before	55.11±8.9	48.58±5.97	47.55±6.4
N=38	After	56.41(7.04)	49.27(7.94)	49.79±8.72
T		0.97	0.59	1.73

N/S not all p values are significant.

The mean score of self-regulated learning pre test- post test was not significant. But the mean of the students’ scores increased after intervention (Table 2).

**Table 2:** Mean Difference in self-regulated learning within groups before & after intervention

Self-regulated learning		T
before	46.50±6.58	0.17
After	48.25±7.68	

N/S not all p values are significant.

The mean score of the self-regulated learning and value of team pre test- post test were statistically significant. These results mean that the students’ scores increased after intervention (Table 3).

**Table 3:** Mean Difference in problem solving and value of team in integrated Web Quest group before & after education

Problem solving skills					Value of team		
	
	Mean	SD	T	P	Mena SD	T	P
Before	1.20	19.80	4.15	0.001	59.85(10.83)	3.33	0.002
After	1.37	1.43			67.08 (6.43)		

Other results regarding students’ satisfaction with the educational method showed the satisfaction of 30 to 90% of the students with the training practices: 65% of students had 80-90% satisfaction rate, 20% expressed a satisfaction rate of 50 to 70%, and the rest expressed it as to be between 30 to 40%.

Other impacts of this program were as follows: on deep learning (67), active and real experience (36), practicality (34), learning and recognition of diseases (12%), not being passive but being student-centered (45%), teacher’sgiving importance to students and spending energy (25%), the appropriate atmosphere of the class and flexibility (53%), a real understanding of the patient and his symptoms (43), creating an interest in students in topics (28%), motivating the students (67%), adaptation with students’ learning (11%), novelty and attraction compared with the traditional approach (a new form of learning) (37%), and the value of training (23%).

## 4. Discussion

This study investigated the effect of WebQuest activity in integrated with case studies and team learning on students’ learning behaviors. Based on the statistical analysis, the mean scores of problem-solving and the value of team work showed a significant difference compared to before the training.

In view of the students ’satisfaction and its close affinity with nursing inreality, Web Quest in nursing education has been considered as a new and valid method for the teaching and learning processes (Pereira, Melo, Silva, & Évora, 2010).

According to March, Drozd and O’Donoghue assert that the most important factor related to the students’ learning in using the Web Quest is how the professors relate technology-based activities to other learning activities (Drozd & O’Donoghue, 2007).

These results are confirmed by other studies: Web Quest’s impact on the development of problem-solving ability, active learning and in relation to content is high (Elwan, 2007; Lim & Hernández, 2007). Schwarz states that Web Quest is a means that provides the students with the opportunity to talk about concepts, to use critical thinking and to solve problems (Schwarz, 2014). Also, in a study by Gülbahar et al. (2010), the students expressed that Web Quest had helped them to learn how to solve real-life problems (Gülbahar, Madran, & Kalelioglu, 2010). In fact, WebQuest enhances students’problem-solving skills because it allows them to expand and refine their knowledge and skills for exploring and integrating new skills. In addition, Web Quest can be designed for students’ learning in collaborative groups, a technique whose success in promoting problem-solving skill has been proven. The nature of Web Quest is that students complete at ask, solve a problem or solve the problem by using a variety of thinking skills, such as comparing, classifying, and generalizing based on the principles of observation and analysis, deduction, exploring the issues, and assessing theirown work (Lahaie, 2008).

With regard to the necessity of problem-solving skills in nursing careers, ranging from education and research to clinical, and due to its undeniable role in the success of nurses in the areas of management, research, education, care, etc., it can be speculated that promoting this skill will improve the performance of nurses in their critical and important duties (Shahbazi & Heidari, 2012). Similarly, Wang (2004) showed that, the more familiar students become with problem-solving strategies, the more successful the application of these skills in clinical care will be (Wang, Lo, & Ku, 2004).

The results of the research by LI et al. (2013) showed that integration of problem-based training in curricula canbe effective in improving the quality and effectiveness of medical education. All ofthe students whowere trainedin this wayhad better result on written exams, physical exams, and had a better overall performance (Li et al., 2013). Another study showed that the students who were more involvedin the educational process and assessment, had substantially better knowledge (McHarg, Kay, & Coombes, 2012). Yu, et al. (2013) also applied problem-based approach in a surgical unit, and realized that problem-based learning was significantly effective in enhancing the ability of critical thinking in nursing students (Yu, Zhang, Xu, Wu, & Wang, 2013).

Nursing educators are more likely to use Web Quests and nursing trainers if they realize that Web Quests are based on educational approaches that are consistent with the principles of learning, so they can incorporate them in their educational activities and use them to create cooperative and group activities that promote social interactions (Lahaie, 2008). In this study, the students were obliged to search the contents needed fortheirown learning and, by resolving the scenarios presented by teachers through doing research and thinking, engage in learning and gain the necessary skills for participating in group work, transferring their knowledge to other students and convincing them.

Regarding the impact of this consolidated approach on the value of teamwork, this educational method had a significant impact on the formation of the value of teamwork; the results of other similar researches confirm this finding. In a study by Cheng, where team training method was used in a maternal and neonatal course for nursing students, TBLmade significant contribution and increased class involvement and the value ofteam work (Cheng, Liou, Tsai, & Chang, 2014).

Also, in a study by Espey on the economics students’ attitude towards teamwork learning, the students had a more positive attitude towards teamwork after the implementation of the method. Interestingly, the poor students in the class were more positive about a strong TBL, while the strong students preferred individual learning methods (Espey, 2010). Likewise, many studies have shown the greater impact of team learning on lower academic percentiles than the upper percentiles (Cheng et al., 2014; Koles, Stolfi, Borges, Nelson, & Parmelee, 2010; Nieder, Parmelee, Stolfi, & Hudes, 2005; Tan et al., 2011). On one hand, this matter can be related to receiving feedback from one’s partners, and on the other hand, to strengthening the skills of reflection and self-improvement in team learning.

But Clark et al., in a study titled “Using team learning in training nursing students”, did not notice any significant difference in the pretest and posttest of the value ofteamwork questionnaire, although students had not a high score regarding the value o team work from the beginning of the study and had not a good attitude towards the value of team work (Clark et al., 2008). Perhaps, the lack of significance in the value of team work in the study of Clark et al. could be attributed to use of different questionnaires. On the other hand, the students experienced ntegrated training in this study, which certainly increases the value of teamwork for the students, compared with teamwork alone.

The research results also showed that the students’scores of self-regulation and self-direction increased after training; however, they were not statistically significant. Learnerswhouse more self-regulation strategies by making the information meaningful, creating a logical connection with the previous data, controlling these processes and creating the appropriate learning environment, while teachers teach or when they study, learn the content and improve their academic performances more successfully (Kajbaf, Molavi, & Shirazi Tehrani, 2003). Also, in Web Quest, learners develop newly acquired knowledge and give meaning to it based on the constructivist theory (Lahaie, 2008). And since this kind of educational environment is an open learning environment, it is necessary for students to have more efficient regulatory skills. This approach, through granting independence to students, provides for higher self-control skills than traditional education does (H. S. Song, Kalet, & Plass, 2011). Klunklin et al. analyzed the experiences of nursing students in problem-solving learning and attributed the improvement in the thinking processtothis method (Klunklin, Subpaiboongid, Keitlertnapha, Viseskul, & Turale, 2011). According to astudy by Noohi et al., students profited from practices of self-esteemand self-interest by using problem-based learning method. Motivating students to think will lead them to use self-regulated learning strategies more, which will in turn enhance learning (Noohi, Abaszadeh, Maddah, & Borhani, 2013). Based onthese results, it is fit and appropriate that teachers provide students with more opportunities for learning by creating conditions that are suitable for the growth of self-development and by teaching self-regulated learning strategies. It is also desirable that they create the right condition s for learners to learn more through changing the teaching methods.

Song and Hill state that learners’level of self-direction may change in different learning contexts (L. Song & Hill, 2007). Mayvng et al. acknowledge that academic resources are those cases that can positively affect students’self-direction (Maung, Abas, & Abdullah, 2007). The effectiveness of the Internet and information technology increating and sustain ingself-direction in learning has been confirmed in several studies (Garrison, 2003; Tondeur, Van Braak, & Valcke, 2007; Watts & Lloyd, 2004). On theother hand, learners’ active involvement inand controlling the learning process is another important matter that can help learners to improve their abilities in order to use resources and strategies effectivel (Vonderwell & Turner, 2005). Sile’n and Uhlin, referring to problem-oriented learning, state that self-directed learning (SDL) is a basic concept in problem-based learning (PBL) and, in a broader sense, plays a central role instudent’s learning (Silén & Uhlin, 2008). Moreover, team learning also provides an environment conducive to self-directed learning and active learning (Tan et al., 2011; Vasan, DeFouw, & Holland, 2008). Because Web Quest was used in the form of problem-based and team learning in this study, obviously, these educational programs must also have affected students’ self-direction. Consideration of students’ backgrounds for accepting learner-based approaches and preparing them are topics that should be treated in more depth.

Another examined variable in this study was students ’satisfaction with the conducted practice: 85% of the students reported their satisfaction o be over 50%. Similarly, in a study where Web Quest was used for teaching nursing students, students reported that they liked the sources of information available on the Web because these sources led to a deeper understanding of the subjects; overall, the students’ impressions of Web Quest was good (Drozd & O’Donoghue, 2007).

In another study, students considered their use of WebQuest as a very good and positive experience in learning and described the possibility to know the points of view of other students as a beneficial aspect of Web Quest (Gülbahar et al., 2010). In a study by Hassanien (2006), the participants viewed Web Quest as a stimulating activity and approximately 90% of the learners recommended that other learners should use theWeb Quest (Hassanien, 2006). In a study by Hassan zadehet al., where team learning was conducted on187medical students at Tehran University of Medical Sciences, over half of the students described team learning as a proper method: many of the students changed their places or stood up right in class in order to be able to participate more ingroup work (Hassanzadeh, Abolhasani, Mirzazadeh, & Alizadeh, 2013). Other researchers who have studied team learning have high lighted such issues as deep learning (Hassanien, 2006; Hassanzadeh et al., 2013), motivating students (Cheng et al., 2014; Clark et al., 2008; Feingold et al., 2008), being learner-based and encouraging students to participatein class (Feingold et al., 2008) as some other benefits of this approach.

## 5. Limitations

One limitation of this study is the small sample size. 38 undergraduate nurses participated in this study. This may not fully allow for generalizing the findings to the entire population of students. Moreover, this study was a quasi-experimental intervention research without a control group. Experiment and comparative study with traditional and active methods of teaching are suggested. Despite these limitations, this research is significant due to the scarcity of studies related to CBL and TBL as integrated methods in the field of nursing education. Also, mixing qualitative and quantitative methods is one of the strengths of this study. Further studies are needed to confirm the benefits of integrated methods for nursing education.

## 6. Conclusion

It is obvious thatour students must have the necessary preparation to confront professional challenges. Using combined teaching and learning methods and strengthening individual land collective potentials can enhance students ’cognitive faculties and learning; therefore, application of integrated methodscan beusedasaneffective approachinmedical education.
